# 

**Published:** 1877-02

**Authors:** 


					Alumni Notice.?We have received the following circular from
Dr. Wm. B. Wise, of Norfolk?the Corresponding Secretary.
Dear Sir :?You are respectfully invited to attend the second
annual meeting of the reorganized "Alumni Asssociation," of
the Baltimore College of Dental Surgery, to be held in the College
Building, on Friday Morning, March 9th, 1877, commencing at
10 A M.
Every graduate of this College is urged to be present, and
participate in the proceedings : also, graduates of other Dental
Colleges are cordially invited.
By order of
Samuel J. Cockerille. of Class of 1853,
President A. Association, Washinqton D. C.
William B. Wise, of Class of 1874,
Corresponding Secy, Norfolk, Va.
P. S.?The 37th Annual Commencement of our Alma Mata
will be'held at the Academy of Music, on the evening of the
same day, March 9th, 1877, to which all the Alumni, and
472 American Journal of Dental Science.
others interested in dental science, are cordially invited. Vale-
dictory Address by Dr. William H. Dwinelle, of New York City.
All graduates will please send their P. 0. address to the Dean.
TO KKADERS AND (J< >KKHSl'ON l>KM n.
Communications intended for publication, and exchange journals and papei
should be addressed to the Editor of the American Journal of Denta!
Science, 259 N. Eutaw St., Baltimore, Md. All letters relating to the busineg
of the journal, or containing cash remittances, to be directed to Messrs. Snow
& Oowman. Articles for publication should be received by the editor ai
three weeks previous to the first day of the month on which the number in w
it is designed for them to appear, is issued.
IPWThe American Journal of Dental Science will contain fifty pasres c,i i
reading matter it] each nti.nber, making a volume of not less than
JSix Hundred pages
EXCLUSIVE OF ADVERTISEMENTS
T H K
AMERICAN JOURNAL
or
DENTAL SCIENCE.
F. J. S. GORGAS, M. D., D. D. S., Editor.
The Journal is issued on the first of each month and contains not less
than fifty pages of reading matter in each number, exclusive of adver-
tisements, making a volume of six hundred pages per annum.
In each number will be found Original Communications, Corres-
pondence, Selected Articles, Monthly Summary, Bibliographical No-
tices and Editorial Matter, and every effort will be exerted to render
the Journal worthy of the patronage of the Dental profession.
A generous co-operation is respectfully solicited from the members
of the profession ; and as the Journal has now a printing office of its
own, which materially lessens the cost of its publication, it will be
issued at the reduced subscription price of
$2.50 IE3ox* Annum in Advance.
Communications are respectfully solicited on all subjects pertain-
ing to the practice of dentistry, as well as reports of cases occurring
in dental practice.
RATES OF ADVERTISING.
1 Page.
i "
J "
1 MO.
$15 00
10 00
7 00
5 00
2 MO.
$22 50
15 00
11 00
8 00
3 MO.
$30 00
20 00
15 00
11 00
6 MO.
$50 00
30 00
20 00
15 00
12 MO.
$100 00
50 00
30 00
20 00
All original communications designed for publication, works for
review, new instruments for notice, exchanges, &c., should be directed
to the editor, 259 N Eutaw St., Baltimore, Md.
All advertisements and subscriptions should be addressed to
SNOWDEN & COWMAN,
Publishers of the Am. Journal of Dental Science.
8&.W. Fayette St. Bet. Charles & Liberty Sts.,
BALTIMORE. MD.

				

